# Unexpected low burden of coronavirus disease 2019 (COVID-19) in sub-Saharan Africa region despite disastrous predictions: reasons and perspectives

**DOI:** 10.11604/pamj.2020.37.352.25254

**Published:** 2020-12-16

**Authors:** Daryl Nzokou Tcheutchoua, Aurel Tiakouang Tankeu, Dominic Leandry Wouna Angong, Batakeh Ba Agoons, Nathan Yves Yanwou Nguemnang, Hugues Clotaire Nana Djeunga, Joseph Kamgno

**Affiliations:** 1 Public Health Department, Faculty of Medical and Paramedical Sciences, Aix-Marseille University, Marseille, France,; 2 Department of Biomedical Sciences, University of Lausanne, Lausanne, Switzerland,; 3 Centre for Research on Filariasis and Other Tropical Diseases, Yaoundé, Cameroon,; 4 Public Health Department, Faculty of Medicine and Biomedical Sciences, University of Yaoundé I, Yaoundé, Cameroon

**Keywords:** Severe acute respiratory syndrome coronavirus-2, COVID-19, sub-Saharan Africa, burden, explanations, reasons

## Abstract

The severe acute respiratory syndrome coronavirus-2 (SARS-CoV-2) is responsible for the development of a highly contagious disease called coronavirus disease (COVID-19). Ten months after the onset of the pandemic, America and Europe remain the most affected regions. Initially, experts predicted that Africa, the poorest continent with the most vulnerable population and health system, would be greatly affected by the ongoing outbreak. However, 240days after the first confirmed case, Africa is among the least affected region, with lower than expected incident cases and mortality. In this review, we discuss possible explanations and reasons for this unexpected low burden of COVID-19 in Africa. We focus on the characteristics of the virus, specificities of the sub-Saharan African population and local environment.

## Introduction

The world is currently facing a highly contagious infectious disease, that originated in China, in December 2019, named coronavirus disease 2019 (COVID-19) by the World Health Organisation (WHO) [1]. This condition is due to the severe acute respiratory syndrome coronavirus-2 (SARS-CoV-2), causing a range of clinical signs including fever, cough, asthenia or respiratory distress [2].

The outbreak was classified by the WHO as a Public Health Emergency of International Concern on January 30^th^, 2020 and was declared a global pandemic on March 11^th^, 2020 [2]. From its first notification in Wuhan (Hubei province, China) on December 31^st^ 2019, the virus rapidly spread throughout the country, then the sub region, and the other continents in less than four months, becoming a major global health concern [2]. The first cases in the American and European regions were reported in United States and France on the 20^th^ and 24^th^ of January 2020, respectively [3,4]. The virus then disseminated very fast in Europe, the number of cases increasing exponentially leading WHO to consider Europe as the novel epicentre of the disease by March 13^th^, 2020. As of October 26^th^, 2020, America became the most affected region with 19,629,999 cases and 624,544 deaths, followed by Europe with 9,472,859 cases and 269,102 deaths [5]. In sub-Saharan Africa (SSA) or WHO African region, the first case was recorded on February 27, 2020 in Algeria [6]. Indeed, the first cases in SSA were mainly imported from Europe followed by a local transmission and the virus spread throughout the continent. As of October 26^th^, 2020, SSA has recorded 1,295,541 cases with 29,191 deaths [5]. These figures in SSA are remarkably low compared to other regions, especially in light of the disastrous projections and warnings from experts [7,8]. Indeed, eight months after the first confirmed case in the SSA, figures are still far below projections and SSA seems to overachieve in the control of this condition with a considerably low evolution of the diseases in this region despite the lack of resources and poor health systems [9]. In this review, we discuss reasons that could explain the low evolution of COVID-19 in SSA and attempt to provide some perspectives.

## Methods

Literature search was performed in PubMed, Web of Science, “Scopus”, “Science Direct” using the following terms: coronavirus, COVID-19 OR SARS-CoV 2 OR 2019-nCoV and Africa OR sub-Saharan Africa to find articles published from January 5^th^ to October 26^th^, 2020. We checked the reference lists of all studies identified by the above methods for cross references. Studies were excluded if they used old data, had inappropriate topics and were not pertinent to the focused purpose of the study. Epidemiologic data were collected from WHO situational reports, and Johns Hopkins Coronavirus Resources Centre.

## Current status of knowledge

### Epidemiology of COVID-19 worldwide

**Figure 1:**
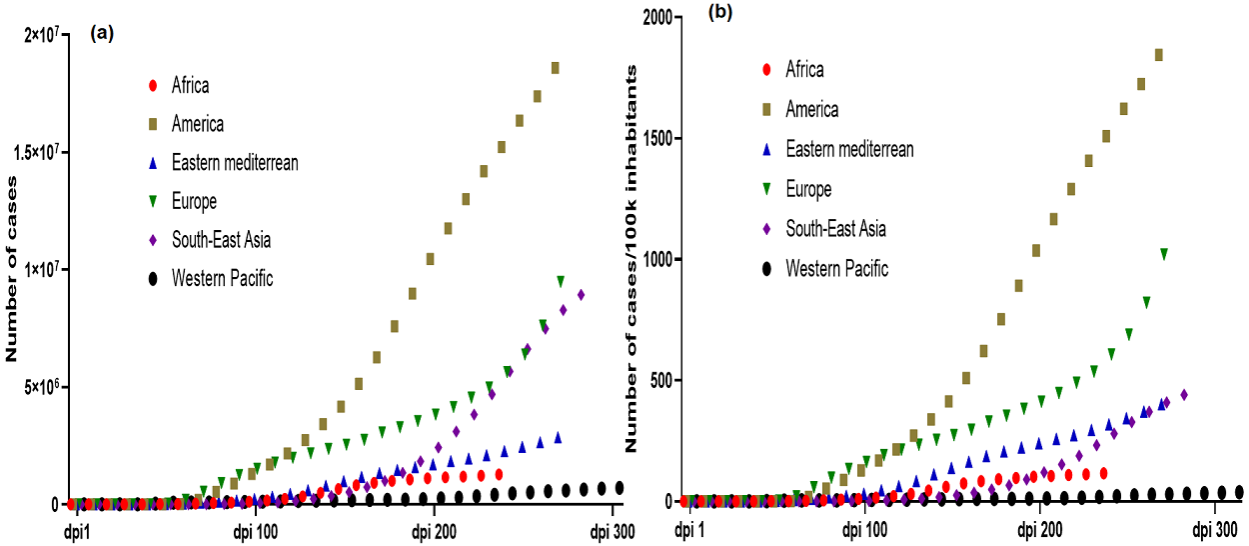
crude (a) and normalized (b) cumulative COVID-19 cases in WHO regions

**Figure 2:**
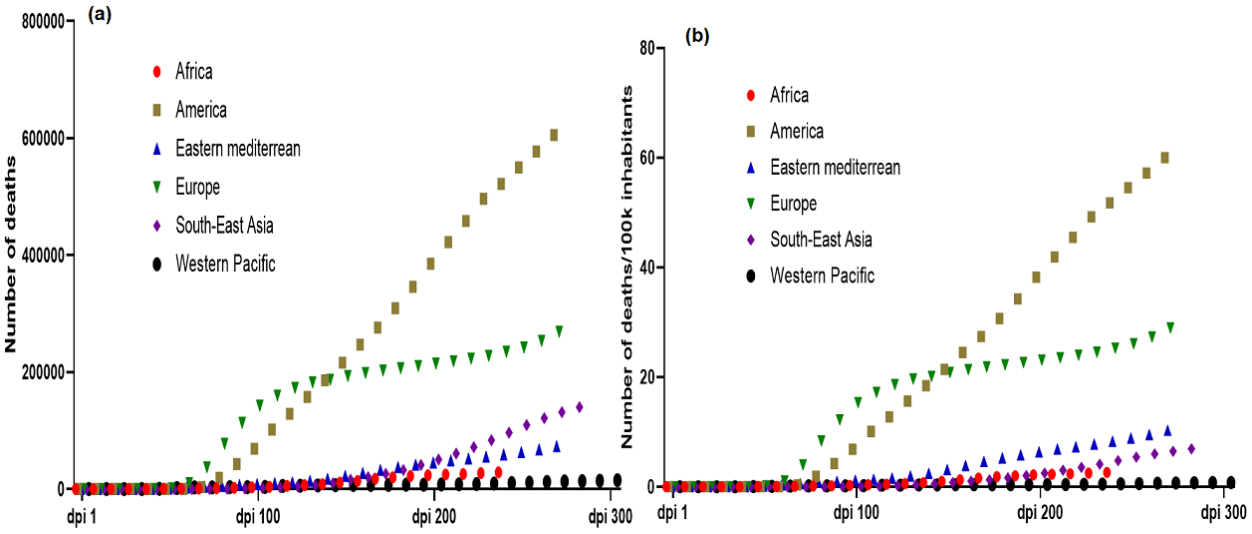
crude (a) and normalized (b) cumulative COVID-19 deaths in WHO regions

**Figure 3:**
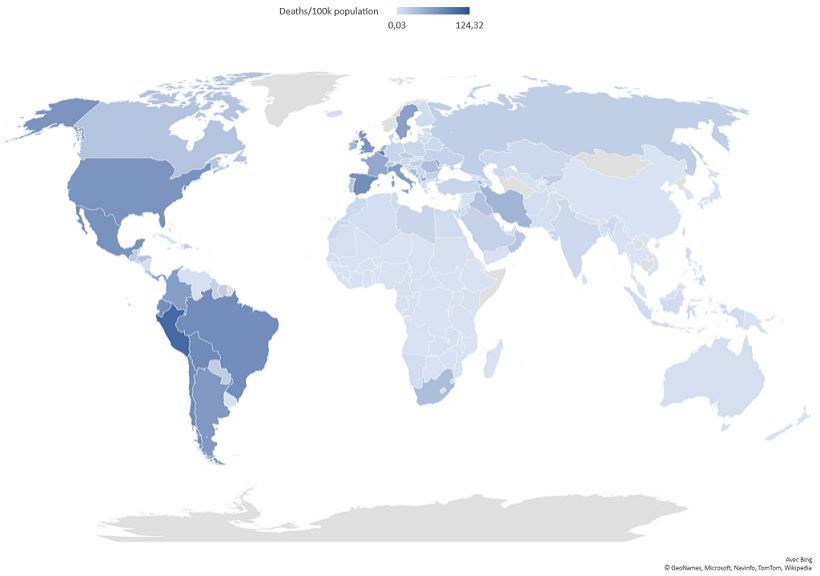
mortality per 100 thousand individuals by country on the 26/10/2020 (Data from Johns Hopkins Coronavirus Resource Center

As of October 26^th^, 2020, COVID-19 disease has affected approximately 42,970,106 people, with 437,683 deaths in 215 countries and territories across the globe [7]. America and Europe are currently the most affected regions, with 19,629,999 and 9,472,859 cases which has resulted in 624,544 and 269,102 deaths, respectively. Surprisingly, SSA remains the least affected region with 1,295,541 cases and 29,191 deaths recorded (Figure 1, Figure 2, Figure 3) [7]. In order to provide a more accurate comparison between regions with different populations and account for the start date of the pandemic (different in each region), we normalized number of cases and deaths to (i) the population of each region and (ii) the number of days following the inception of the pandemic referred here as the number of days post-inception (DPI). This comparison clearly revealed that until now, the evolution of cases and deaths in SSA is much lower than in Europe and America, whatever the metric used. Furthermore, we normalized the number of deaths to the population and the DPI rather than the number of confirmed cases or the case fatality rate, to take into account that the number of cases is highly dependent of the number of people tested. By October 26^th^, 2020, the DPI was 245 in WHO African region, 278 in America, 273 in Eastern Mediterranean region, 270 in Europe, 280 in South-East Asia and 300 in West pacific region. The comparison of the epidemiological data is shown in Table 1.

**Table 1:** cumulative COVID-19 deaths/100 thousand individuals normalized to DPI

Cumulative deaths/100k individuals	DPI 1	DPI 20	DPI 40	DPI 60	DPI 80	DPI 100	DPI 120	DPI 130	DPI 130 deaths ratio (region/SSA)
**Africa**	0	0.0003	0.0216	0.1520	0.1487	0.2618	0.4847	0.6072	/
**Americas**	0	0	0	0.0250	1.9131	6.8506	12.7567	15.6375	x 26
**Eastern Mediterranean**	0	0	0.0209	0.3521	0.7949	1.1884	1.6078	1.9689	x 3.2
**Europe**	0	0	0.0041	1.0974	8.3384	15.3658	18.6283	19.6470	x 32
**South-East Asia**	0	0	0	0.0015	0.0231	0.0989	0.2362	0.3382	x 0.55
**Western Pacific**	0	0.0003	0.0433	0.1522	0.1811	0.2098	0.3212	0.3430	x 0.56

### Public health control policies against COVID-19

With the sudden nature of the pandemic, pharmacological treatment and vaccine against the COVID-19 disease are yet to be approved; the major control strategies therefore aimed at slowing the spread of the disease, so as to avoid the overburdening of health systems [10]. Through the suppression strategy, comprising physical distancing, household quarantine, school and university closures, and home isolation of non-severe cases was largely adopted, its policy was also largely country dependent. Indeed, in Europe, the Italian government imposed a national quarantine, restricting the movement of its citizens except for essential needs [11]. Several other countries such as France, Spain, Switzerland, Germany also proceeded with a national lockdown and closure of all public spaces and borders except for special cases [12]. The exact duration of the lockdown also varied from one country to another but lasted about two months in most cases. In North America, the suppression strategy was implemented on the 2^nd^ of February 2020, with the United States of America prohibiting entry of travellers with a recent travel history to china. South American countries such as Brazil followed by suspending flight arrivals from case confirmed regions, and countries such as Mexico, Peru and Argentina [13]. However, despite its proven efficiency in lowering the spread of the pandemic, the lockdown led to devastating economic impact, the severity being associated with the length of the lockdown [14]. Therefore, some countries such as South Korea adopted a different approach which, aimed to screen as many individuals as possible, isolating confirmed cases only, thus maintaining almost unchanged the country´s functioning [15]. Similarly, Singapore implemented restrictive measures without total lockdown keeping schools and businesses opened with precautionary measures such as regular temperature checks. Rather than suppression or mitigation strategies, herd immunity, consisting of acquired immunity by the majority of population either through infection or vaccination was experimented by some countries such as Sweden [16]. The strategy aimed to allow the spread of the disease amongst active and lower risk population while preserving, in isolation, high risk population (older persons and those who have chronic illness) [16].

SSA region is mostly composed of low- and middle-income countries with very limited resources. As such, populations rely on daily activities for living. Therefore, a complete lockdown, especially businesses closure, represents a significant challenge and even appears unrealistic. Most SSA governments have implemented measures to (i) encourage physical distancing, focusing on border and travel restrictions, school closures, and bans on large gatherings; (ii) promote masks wearing and frequent hand washing, while testing suspected cases, tracking their contacts, and treating confirmed ones [17,18]. The first country to implement a lockdown in the WHO African Region was Rwanda on the 21^st^ of March followed by South Africa, which implemented the strictest lockdown in SSA. Other countries, such as Senegal, Ghana, Cameroon and Ivory Coast, instituted curfews, and partial lockdowns. Countries like Burundi instituted a 14-day quarantining for people entering from affected countries [19].

### Potential reasons for the lower than expected COVID-19 burden in Africa

The spread of infectious diseases usually results from the interaction between three main factors: the infectious agent, the host´s characteristics and the environment [20]. Therefore, we built different hypotheses that can explain the lower burden of COVID-19 disease in sub-Saharan Africa focusing on these three parameters.

### SARS-CoV-2 virus strains

The pathogen responsible of the COVID-19 disease is the SARS-CoV-2; a positive sense single stranded RNA virus. Such viruses are characterised by a remarkable ability to adapt to new hosts and environments through mutations in a short time period [21]. These mutations can be neutral or lead to recombination, creating slightly different virus strains/variants. In an early analysis of SARS-CoV-2 genomes from 160 cases, Forster and colleagues distinguished three central variants: (i) the ancestral variant A, predominantly found in early infections from China and East Asia neighbouring countries as well as the first samples from United States and Australia [22]; (ii) the type B variant predominantly isolated from samples of eastern China and neighbouring Asian countries, and rarely outside of East Asia, as well as from the first European samples from France, Germany, and Italy; (iii) the type C variant was the major European type. Very little is known about the variant circulating in Africa since this region accounts only for 1% of the total number of genome sequences in the global SARS-CoV-2 genomic database [23]. Since most African infections were imported from Europe, the circulating variant in SSA might be close to the European types. However, we cannot exclude the fact that after several months, the SARS-CoV-2 strain currently circulating in SSA can be slightly different from initial variants. Nevertheless, this probable change in viral genome is less likely to explain the differences in evolution of the pandemic between SSA and western regions. On the other side, SSA was among the last regions affected and few days after confirmation of first cases, most of the countries closed their borders, therefore isolating themselves and the region from the rest of the world. Unlike Africa, western countries left their borders opened for many weeks before isolating themselves and their respective regions from the world. This may have allowed multiple importations of virus strains from different regions of the world, explaining why more than one circulating SARS-CoV-2 variant is found in these western countries. Despite the scarcity of information, we cannot rule out the possibility that the presence of multiple variants in the same setting can promote recombination resulting in viral virulence differences. Phylogenetic studies are therefore highly needed to investigate the SARS-CoV-2 strains circulating in Africa and assess their genetic difference with other strains with regards to their virulence since the first ones were done before the arrival of the virus in Africa [22].

### Demography: age structure

Early reports from China pointed out age as the most important risk factor for COVID-19 disease mortality, with case fatality rate increasing from <0.5% for individuals aged <50 years old to 3.8% between 60-80 years old, and up to 14.8% for those aged >80 years [24]. Similar trends were observed in the United States, with the case fatality rate ranging from <1% below the age of 50, to 10.4 - 27.3% above the age of 85 years [25]. This susceptibility among the elderly may be explained by the occurrence of co-morbidities that worsens with age [26]. In addition, muscle atrophy, changes in the anatomy of the lungs that lead to a reduction in respiratory volumes are frequent, making the elderly more at risk of respiratory failure [27]. The median age in SSA is 19.7 versus 43.1 years in Europe and 38.6 years in North America. In addition, only 3% of the population is over 65 years old compared to about 20% and 16% in Europe and North America, respectively [28]. The younger age of the African population might act as a protective factor against COVID-19, thus explaining the low mortality observed in SSA. However, this fatality rate related to the age does not explain why there are less infected subjects in Africa. Since testing policy was based on population complaints, it seems likely that the proportion of asymptomatic careers is high because of the tolerance of the disease by young people.

### Co-infection patterns

African environment is characterised by multiple infections with wide range of viruses, bacteria, and parasites because of the tropical climate and poor sanitation, which are favourable to the proliferation of these infections. It is known that helminths can modulate immune response of their hosts; indeed, infections are usually chronic and can even persist for several years corresponding to the natural life expectancy of these parasites in the absence of treatment. The immune response to helminths is therefore relative, with the host tolerating these parasites which then migrate into the connective tissue, causing damage to the host via several mechanisms as part of active immunomodulation [29]. Indeed, the ability of parasites to challenge host immunity is achieved through the release of a spectrum of finely tuned and highly advanced immunomodulatory factors, likely inducing tolerance to other infections. Since these immunomodulation-based infections are highly prevalent in Africa, and considering that symptomatic and severe COVID-19 cases were associated with a cytokine storm, it is likely that these parasites may have played a role in the virulence of the virus, thus resulting in high number of asymptomatic cases.

### History of tuberculosis vaccination

**Figure 4:**
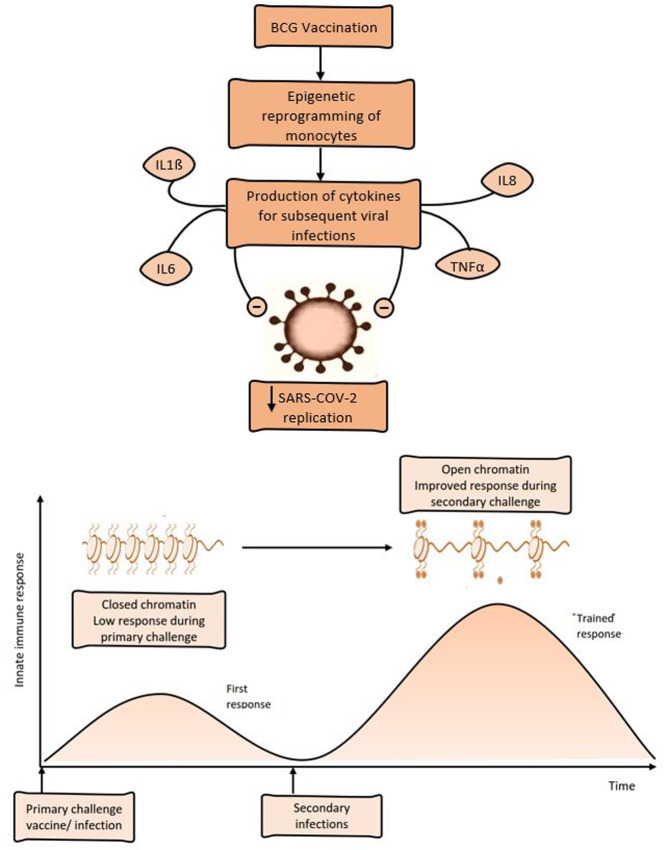
trained immunity antiviral host defense (adapted from O´Neil et Netea, 2020)

Tuberculosis (TB) is known to be highly endemic in low resource settings, with most cases being found in South-East Asia (44%) and Africa (24%) while the lowest figures are reported in America (3%) and Europe (3%). TB incidence is 226 per 100,000 in South-East Asia and 237 per 100,000 in SSA, accounting for nearly 70% of the global TB cases [30]. In these high-incidence countries/regions, routine Bacillus Calmette-Guerin (BCG) vaccination is recommended by the WHO immediately after birth [31]. Thus, BCG is part of many national immunization programs in SSA countries to ensure optimal coverage of the entire population. In 2018, about 80% of SSA individuals received BCG vaccination [32]. Evidence from randomized controlled trials reveals that the BCG vaccine can protect against respiratory infections and reduce death from pneumonia as a consequence of its immunomodulatory properties [33]. This off-target effect on the immune system could promote a protection from severe pneumonia due to SARS-CoV-2 in vulnerable groups. Indeed, BCG has heterologous beneficial against non-related infections by a genome-wide epigenetic reprograming of monocytes, resulting in an upregulation of cytokines such as IL-1ß [34]. Apart from monocytes, BCG can induce a long-lasting heterologous T helper 1 and T helper 17 immune response and an increased production of TNFa, IL-1ß and IL-6 to mycobacteria and unrelated pathogens [35]. It is possible that this trained immunity could protect against SARS-CoV-2 (Figure 4). An epidemiological study comparing COVID-19 outcome between countries without a universal BCG vaccination policy and countries with a current universal vaccination policy demonstrated that BCG vaccination could significantly reduce mortality associated with COVID-19. Furthermore, the study revealed that the earlier a country established a BCG vaccination policy, the highest the reduction in the number of COVID-19 deaths per million inhabitants [36]. Another study revealed that children who have been vaccinated with BCG are less susceptible to COVID-19 and therefore less likely contribute to the spread of the virus to older and at-risk populations [37]. Thus, the universal vaccination policies against tuberculosis implemented in SSA could explain the low mortality rate observed so far. However, there is no evidence that elderly people maintain a sufficient pool of trained monocytes several years after vaccination, and further clinical studies are required to validate this hypothesis.

### Traditional medicine to complement poor health system

In a bid to cure many diseases (including COVID-19) in SSA, there is regular herb consumption, and recourse to traditional medicine. Eighty percent (80%) of SSA population is estimated to have ever used traditional medicine, according to WHO. Indeed, in the absence of well-organized/structured health systems, the first therapeutic recourse against the COVID-19 pandemic was traditional medicine. Frequent consumption of these medicinal plants might have influenced the course of this pandemic in SSA, resulting in lower mortality compared to other regions. Studies showed that SARS-CoV-2 infection is associated with oxidative stress contributing to pulmonary fibrosis [38]. Several local plants such as *Zingiber officinale, Allium sativum, Panax ginseng, Eucalyptus globulus*, and *Artemisia annua* exhibit antioxidants and immunomodulatory properties, and are consumed across the continent in various forms and preparations for prevention or treatment of several conditions [39]. For example, 6-Gingerol, a bioactive compound of *Zingiber officinale*, shows highest binding affinity and interaction with multiple targets of COVID-19 including viral proteases, RNA binding protein and spike protein [40]. A Chinese study revealed that ginsenoside-Rb1 (isolated from panax ginseng) and extract of eucalyptus globulus inhibit SARS-CoV-2 replication [41]. Also, an antiviral activity against SARS-CoV-2 was identified in compounds extracted from *Artemisa annua* [42].

### Ivermectin - the wonder drug

Ivermectin is widely used in SSA for the treatment of various Neglected Tropical Diseases (NTDs) including onchocerciasis and lymphatic filariasis. Inhibitory activity of ivermectin on the replication of some single-stranded RNA viruses such as dengue virus, Zika virus or yellow fever virus is largely documented and it´s actually considered as a possible drug for SARS-CoV-2 [43]. Indeed, by inhibiting the viral IMPa/ß1-mediated nuclear import, ivermectin can reduce the SARS-CoV-2 viral load [44]. Two recent clinical trials ivermectin-doxycycline (n=60) VS Hydroxy chloroquine-Azithromycin (n=56) and ivermectin (n=173) VS standard of care (n=107) found a better success on symptoms relief, a reduction of the recovery duration and a lower mortality in the ivermectin treated group [45,46]. However, a pharmacokinetic report estimated that the doses routinely used for the control of parasitic diseases are less likely to lead toSARS-CoV-2 inhibition [47]. Results of larges randomized controlled clinical trials ongoing are awaited to better understand the possible effect of routine ivermectin in SSA. In 2019, nearly 212 million people were treatment with ivermectin in Africa and mainly in rural areas where the virus spread the least.

### Genetic differences between populations

The genetic background of populations is known to modulate the susceptibility and severity of many diseases. Based on the delayed infection of SSA individuals and the low prevalence in the African-American communities in the early weeks of the pandemic, it was initially suggested that the genetic background of SSA individuals could render them less susceptible to COVID-19 [48]. A difference in expression of angiotensin converting enzyme 2 receptor was evoked to support this hypothesis [49]. With the evolution of the pandemic, COVID-19 mortality increased among African Americans compared to the other ethnic groups in the United States, likely as a consequence of current socioeconomic disparities and lifestyle leading to more important burden of any health condition on minorities with limited health care access [50].

### Population density in Africa

One of the major differences between SSA and other parts of the world is the population density. Indeed, SSA population is estimated at 1.3 billion habitants, equivalent to 16.7% of the world´s population. With a total area of 29,648,481 Km^2^, the population density in SSA is around 50 habitants/Km^2^ (Hbt/Km^2^). This is less than half of population density in Europe (~105 habitants/Km^2^) [51,52]. Moreover, countries with higher deaths/100,000 population such Belgium, Spain, Italy, United Kingdom and France are those with the high population densities, estimated in 2018 at 377.2 hbt/Km^2^, 93.5 hbt/Km^2^, 205.5 hbt/Km^2^, 274.8 hbt/Km^2^, and 122.3 hbt/Km^2^, respectively [53]. In addition, populations in Europe are concentrated in urban areas with only 24.3% of the population living in rural areas compared to 59.8% in Africa [54]. The high concentration of individuals in urban areas can explain the important transmission in western world especially in populated towns as it was further observed in New York. Indeed, the basic reproduction number (R0), which reflects the transmission of the virus in a population, is estimated around 2-4 for SARS-CoV-2 and depends on several factors including population density [55]. In the Diamond Princess, the density of population in a cruise ship infected by SARS-CoV-2 and quarantined in Yokohama in February 2020 was around four times higher than in Wuhan, leading to about four time increase in the R0 before the beginning of physical distancing measures [56]. Mathematical modelling of a previous respiratory epidemic, Middle East Respiratory Syndrome-Coronavirus (MERS-CoV), demonstrated that population density exhibited a significant effect on the spread of the disease [57]. Even in SSA, the cases are concentrated in urban areas where the highest population density is found. This population concentration promotes contact between individuals and makes it difficult to follow distancing measures, increasing the risk of spreading the virus. Therefore, the overall low population density in SSA might constitute an important driver of the slow evolution of the pandemic in the region.

### Population movements

Mobility in and across countries and regions is a major determinant of the spread of infections, by disseminating the virus out of its epicentre and amplifying contacts between individuals within the same country, between countries and from one continent to another [58]. For instance, regions such as the Ile de France and northern Italy, which were among the first affected in Europe have regular flight exchanges with China especially with Wuhan, the initial epicentre of the pandemic [59]. Mulhouse town, which was the venue of an international religious convention attended by nearly 2,300 worshippers from different parts of the world in mid-February was the starting point of one of the main outbreaks of the epidemic in France [59]. In terms of regional and national movements, there is little movement within SSA countries due to lack of road, rail, and flight transport between different towns of the same country. This is worsened by limited financial resources of the populations to afford cost of these transports when existing and the almost non-existent free movement -as opposed to Europe for example- which further limits interactions between the populations of different SSA countries. African Union figures report only 19 million people who have migrated across Africa (1.6% of the African population) and 17 million people to other continents (1.4% of the African population) [60]. Unlike Africa, movements between European countries are more important. Indeed, a 2012 European Union study reported about 17.8% of the European Union population changing country of residence [61]. Apart from permanent movements, tourism is very developed in western countries, unlike in Africa. About 60% of European citizens travelled for holidays in 2013, 47.3% of them in the same country and 29.5% to a foreign country [61]. Thus, the difference observed between the movements of Africa and other regions´ populations might explain the relatively low number of cases in SSA.

### Fluctuations in temperatures

From the very beginning of the epidemic, climate has been suspected as a factor that could slow or even prevent the outbreak of the epidemic. As with the Influenza virus, a progressive attenuation of the epidemic was predicted with the arrival of summer [62]. However, a study conducted in Jakarta (Indonesia) from January to March 29^th^, 2020 revealed a positive correlation between temperature and the number of COVID-19 cases [63]. Another study conducted in 224 cities in China showed no significant association between ambient temperature and R0, suggesting that temperature has no effect on virus transmission [64]. However, these studies evaluated only small variations in mean temperature over a few months, with a minimum of 26.1°C and a maximum of 28.6°C in Jakarta and a mean temperature of 5.9 +/- 7.5°C in China [63]. These temperature differences are very small compared to that existing between the regions of Africa or South America and Europe or North America. More than five months after the start of the epidemic, the most severely affected countries are mainly in the northern hemisphere (Figure 3), raising once again the question of a possible effect of ambient temperature and/or humidity on the spread of SARS-CoV-2. A study conducted in China revealed that under lower temperature, every 1°C increase led to an increase of the cumulative number of cases by 0.83. In the higher temperature group, every 1°C increase in the minimum temperature led to a decrease of the cumulative number of cases by 0.86 [65]. Another study conducted in China revealed that for temperatures below 3°C, an increase in temperature of 1°C is associated with a 4.9% increase in the number of daily cases [66]. These data suggest an optimal temperature for the spread of SARS-CoV-2 similar to that of SARS-CoV-1 (16 to 28°C) and limited spread of the virus at low or high extreme temperatures [67]. Regarding air humidity an increase in 1% relative humidity lowered by 0.0224 the R0 [68]. Various mechanisms are investigated to explain this decrease in R0 at high temperatures, including the susceptibility of the virus, the low resistance on external surfaces and also the production of vitamin D, which is believed to stimulate immunity against viral infections [69,70]. However, Brassey *et al*. estimated that temperature and humidity alone would contribute a maximum of 18% of virus transmission and cannot stops its spread [71].

### Different management of the epidemic by sub-Saharan African countries

Due to the low trade between Africa and China, Europe has been the main source of COVID-19 imports into Africa. A delay of about 3 to 5 weeks between the emergence of SARS-CoV-2 in Europe and Africa, allowed these countries to prepare for the arrival of the virus [72]. In addition, several African countries implemented measures at a relatively early stage to limit the importation of cases and the early detection of suspected cases. The closure of the Schengen area was announced on March 17^th^ with 64,188 cases and 3,108 deaths of COVID-19 [73]. At the same time, with 228 cases in Africa and 4 deaths, several African countries were already implementing epidemic mitigation measures [7]. Thus, on March 18^th^, and with case numbers per country below 50, several African countries had already put in place measures such as the stopping air and/or sea communication and exchanges with others regions, travel restrictions, closure of public places and 14 days of systematic quarantine for passengers coming from areas where the virus was already circulating [19]. Additionally, experience of previous epidemics such as Ebola and cholera likely increased the preparedness of many SSA countries [74]. As such, governments and communities in these countries were likely better equipped to quickly and effectively respond to new epidemics such as the COVID-19 [9]. For instance, Democratic Republic of Congo, Liberia, Guinea and Sierra Leone recently struck by Ebola epidemic remain among the less affected countries by the COVID-19 in the region [75]. In addition to epidemics, wars, and humanitarian disasters that have occurred in Africa have likely improved the resilience of African populations, leading to better acceptance and compliance with restrictive epidemic mitigation measures.

## Conclusion

This review aimed to compare the progression of the COVID-19 pandemic in SSA and other regions of the world, identify and discuss potential hypotheses that could explain low progression of this condition in the SSA region unlike the disastrous predictions. It appears that different factors inherent to the SARS-CoV-2, the SSA population and the local environment might have influenced the evolution and spread of the pandemic as well as its mortality. However, these factors do not weight the same, variables related to the environment appearing to be the most important. Further studies are needed to analyse the effects and contribution of each of these variables on the COVID-19 in SSA.

### What is known about this topic

- COVID-19 cases and deaths in sub-Saharan Africa (SSA) are lower than predicted.

### What this study adds

- The comparison of epidemiological data on COVID-19 between SSA and other regions;
- Possible explanation of the lower than expected burden of COVID-19 in SSA.
